# It's Not Healthy Fellowship: Negative Interactions and the Implications for Middle- and Old-Age Black Men

**DOI:** 10.1093/geroni/igab046.1631

**Published:** 2021-12-17

**Authors:** Antonius Skipper, Loren Marks, Cassandra Chaney

**Affiliations:** 1 Georgia State University, Atlanta, Georgia, United States; 2 Brigham Young University, Provo, Utah, United States; 3 Louisiana State University, Baton Rouge, Louisiana, United States

## Abstract

Despite the benefits of social support on the well-being of Black men across the life course, scholars are more closely examining the potentially negative outcomes associated with some social networks. As one social support system, the Black church frequently serves middle and old age Black men who identify as religiously involved. Yet, higher levels of religious involvement have also been associated with more church-related negative interactions. The present study utilizes a grounded theory approach to examine the negative interactions of religious middle and old age Black men. A semi-structured interview protocol is used to gather data from 35 Black men between the ages of 45 and 76. Analyses reveal that church-related negative interactions broadly fall within the following themes: (1) Ageism Within Intergenerational Churches, (2) People are Messy, and (3) Issues with Leadership. Since negative interactions can be more detrimental than social support is beneficial, health-related implications are discussed.

